# Evaluation of the clinical and prognostic importance of infection parameters in thyroid cancers: A cross-sectional study

**DOI:** 10.1097/MD.0000000000036532

**Published:** 2023-12-08

**Authors:** Seval Müzeyyen Ecin, Deniz Gezer

**Affiliations:** a Unit of Occupational Diseases and Internal Medicine Clinic, Mersin City Training and Research Hospital, Mersin, Turkey; b Unit of Internal Medicine Clinic, Mersin City Training and Research Hospital, Mersin, Turkey.

**Keywords:** inflammation markers, lymphocytes, neutrophils, platelets, thyroid cancers

## Abstract

Thyroid cancers are among the most common endocrine cancers. An inflammation is associated with many stages of cancer. Therefore, in this study, we aimed to evaluate whether it has a prognostic significance inflammation marker. Neutrophil/lymphocyte ratio, platelet/lymphocyte ratio, monocyte/lymphocyte ratio, systemic inflammation response, systemic immune-inflammation index, and neutrophils to lymphocytes and platelets ratio (N/LP) in patients diagnosed with thyroid cancer in the internal medicine outpatient clinic and operated between March 1, 2017 and May 1, 2022 were evaluated retrospectively. Three hundred forty patients were diagnosed with thyroid cancer; 275 (80.9%) of them were women and the mean age was 44.6 ± 13.5 years. Multifocality (*P* = .02) was significant in patients with invasion. High N/LP ratio (odds ratio: 1.4, 95% confidence interval: 1.0–2.0, p: 0.003) and high invasion (odds ratio: 0.2, 95% confidence interval: 0.1–0.4, *P* < .01) was found to be significant in patients with tumor size ≥2 cm. There is a relationship between multifocality and invasion, and the risk of invasion increases as the tumor size increases in thyroid cancer. The N/LP ratio was significant as it could be a new marker in showing the relationship between thyroid cancer and its prognosis. Further studies are needed in which the prognosis is followed up, longer-term, more comprehensive, and confounding factors are excluded.

## 1. Introduction

Thyroid cancers are among the most common endocrine cancers and account for 96% of endocrine cancers and 3% of all cancers.^[1]^ Although the malignancy risk is low and the prognosis is good, it can also be seen in lymph node and distant metastases that can cause death.^[2,3]^ It has also been shown that inflammation is associated with many stages of cancer such as onset, transformation to malignancy, invasion, and metastasis.^[4]^ Recent studies have supported that inflammation is associated with the initiation and progression of thyroid cancer and inflammation indicators such as neutrophil/lymphocyte ratio (NLR), platelet/lymphocyte ratio (PLR), and monocyte/lymphocyte ratio.^[5–9]^

Therefore, in this study, we aimed to evaluate whether it has a prognostic significance inflammation marker; NLR, PLR, monocyte/lymphocyte ratio, systemic inflammation response (SIRI), systemic immune-inflammation index (SII), and neutrophils to lymphocytes and platelets ratio (N/LP) in patients diagnosed with thyroid cancer in the internal medicine outpatient clinic between 2017 and 2022.^[10–12]^

## 2. Materials and methods

It is a cross-sectional study in which the data of patients aged ≥18 years who were diagnosed with thyroid cancer in the internal medicine outpatient clinic and operated between March 1, 2017 and May 1, 2022 were evaluated retrospectively. Age, gender, tumor type, tumor size, multifocality, presence of invasion, hemogram (hemoglobin, hematocrit, white blood cell, neutrophil, monocytes, lymphocyte, platelet), thyroid stimulating hormone, free triiodothyronine, and free thyroxine were analyzed. Patients aged ≥18 years, diagnosed with thyroid cancer and operated, and who had blood parameters within 1 month before the operation were included in the study. Patients who did not have blood parameters for >1 month were excluded from the study. The sampling time for all patients was between 8 and 12 in the morning. NLR was calculated as the absolute neutrophil count divided by the absolute lymphocyte count; PLR was calculated as the absolute platelet count divided by the absolute lymphocyte count and lymphocytes to monocytes ratio (LMR) was calculated as the absolute lymphocyte count divided by the absolute monocytes count.^[7–9]^ And SIRI was defined as “neutrophil × monocytes/lymphocyte”; SII was defined as follows “platelet × neutrophil/lymphocyte.” N/LP was calculated “(neutrophil × 100)/(lymphocyte × platelet).”^[11,12]^ Tumor size was divided into 2 groups as <2 cm and ≥2 cm.^[13]^

### 2.1. Statistics

Data were calculated using the statistical program SPSS 21.0 (IBM Corp., Armonk, NY). For data not normally distributed, as determined by the Kolmogorov–Smirnov, median ± SD (standard deviation) was used to define numerical variables, and categorical variables were defined as numbers and percentages. Between-group comparisons were performed with the *t* test and the Mann–Whitney *U* test for continuous variables, and the chi-squared or Fisher exact tests for categorical variables as appropriate. Univariate and multivariate logistic regression were used to compare variables of age, gender, N/LP, invasion, and tumor size.

### 2.2. Ethics

Ethical approval given by the Ethics Committee of Non-Interventional Clinical Studies of the Mersin University Hospital approved the study (Protocol No.: 2022/415).

## 3. Results

Between 2017 and 2022, 340 patients were diagnosed with thyroid cancer; 275 (80.9%) of them were women and the mean age was 44.6 ± 13.5 years. The most common cancer in 90.9% of the patients was papillary thyroid cancer, mean tumor size was 1.3 ± 0.8, 80.0% of cases had invasion, and 34.1% had multifocality. Demographic and laboratory data of the patients with thyroid cancer are given in Table 1.

**Table 1:** Demographic and laboratory data of the patients with thyroid cancer.

Features	N = 340
	n (%)
Gender	
Female	275 (80.9)
Male	65 (19.1)
Age, mean ± SD	44.6 ± 13.5
Tumor type	
Papillary	309 (90.9)
Follicular	10 (2.9)
Medullary	17 (5.0)
Hurtle	2 (0.6)
Anaplastic	2 (0.6)
Tumor size, mean ± SD	1.3 ± 0.8
Tumor invasion	272 (80.0)
Multifocality	116 (34.1)
Neutrophil (×10^3^/μL), mean ± SD	4.5 ± 1.9
Lymphocytes (×10^3^/μL), mean ± SD	2.2 ± 0.7
Monocytes (×10^3^/μL), mean ± SD	0.4416 ± 0.2
Platelet (×10^3^/μL), mean ± SD	275.4 ± 73.5
TSH (μIU/mL), mean ± SD	1.9 ± 2.3
T3 (pg/mL), mean ± SD	3.3 ± 1.3
T4 (ng/dL), mean ± SD	1.2 ± 0.7

T3= triiodothyronine, T4= thyroxine, TSH = thyroid stimulating hormone.

In our study, female gender (*P* = .02), high N/LP ratio (*P* = .03), and high invasion (*P* < .01) was found to be statistically significant in patients with tumor size ≥2 cm. And high multifocality (*P* = .02) was significant in patients with invasion. The relationship between tumor size, invasion, multifocality, and age, gender, and systemic inflammatory markers are given in Table 2.

**Table 2:** The relationship between tumor size, invasion, multifocality, and age, gender, and systemic inflammatory markers.

Features	Tumor size			Invasion			Multifocality		
	<2 cm N: 271	≥2 cm N: 69	*P* value	Yes N: 68	No N: 272	*P* value	Yes N: 116	No N: 224	*P* value
Female	226 (83.4)	49 (71.1)	**0.02**	43 (63.2)	232 (85.3)	**<0.01**	95 (81.9)	180 (80.4)	0.7
Age	44.6 ± 13.1	44.7 ± 14.9	0.9	43.5 ± 13.9	44.8 ± 13.4	0.5	46.8 ± 13.2	43.5 ± 13.5	0.06
NLR	2.2 ± 1.2	2.9 ± 3.3	0.1	2.7 ± 2.9	2.2 ± 1.5	0.2	2.3 ± 1.2	2.4 ± 2.1	0.9
LMR	5.6 ± 2.0	5.4 ± 2.0	0.3	5.5 ± 2.4	5.7 ± 2.9	0.2	5.4 ± 1.9	5.7 ± 2.1	0.3
PLR	137.1 ± 59.8	145.4 ± 51.7	0.07	145.7 ± 64.1	136.9 ± 56.7	0.5	0.1 ± 0.1	0.1 ± 0.1	0.3
SIRI	959.1 ± 758.7	1227.2 ± 1605.9	0.4	1178.3 ± 1356.5	972.3 ± 878.5	0.3	974.1 ± 814.7	1034.0 ± 790.1	0.8
SII	615.3 ± 418.0	751.9 ± 751.4	0.3	715.4 ± 671.4	624.9 ± 454.4	0.3	654.8 ± 479.0	641.6 ± 538.9	1
N/LP ratio	0.8 ± 0.5	1.2 ± 1.6	**0.03**	1.1 ± 1.4	0.9 ± 0.7	0.1	0.9 ± 0.5	0.9 ± 1.1	0.7
Invasion	37 (13.7)	31 (44.9)	**<0.01**	**–**	**–**	**–**	34 (29.3)	34 (15.2)	**0.02**

Significant *P* values are indicated in bold.

LMR = lymphocytes to monocytes ratio, N/LP ratio = neutrophils to lymphocytes and platelets ratio, NLR = neutrophil to lymphocytes ratio, PLR = platelet to lymphocytes ratio, SII = systemic immune-inflammation index, SIRI = systemic inflammation response.

In the corrected for age, gender, and invasion multivariable logistic regression analysis, high N/LP (odds ratio, OR: 1.4, 95% confidence interval, CI: 1.0–2.0, p: 0.003) and high invasion (OR: 0.2, 95% CI: 0.1–0.4, *P* < .01) was found to be significant in patients with tumor size ≥2 cm. The adjusted univariable and multivariable logistic regression analysis according to tumor size ≥2 cm in thyroid cancers are given in Table 3.

**Table 3:** The adjusted univariable and corrected for age, gender, and invasion multivariable logistic regression analysis according to tumor size ≥ 2 cm in thyroid cancers.

Features	Univariable		Multivariable	
	Tumor size ≥ 2 cm			
	OR (95% CI)	*P* value	OR (95% CI)	*P* value
Age	0.9 (0.9–1.0)	0.9	0.9 (0.9–1.0)	0.9
Gender	0.4 (0.3–0.9)	**0.02**	0.7 (0.4–1.4)	0.3
N/LP ratio	1.4 (1.0–2.0)	**0.03**	1.4 (1.0–2.0)	**0.03**
Invasion	0.2 (0.1–0.4)	**<0.01**	0.2 (0.1–0.4)	**<0.01**

Significant *P* values are indicated in bold.

CI = confidence interval, N/ LP ratio = neutrophils to lymphocytes and platelets ratio, OR = odds ratio.

## 4. Discussion

Thyroid cancers are the most common type of cancer among endocrine cancers, and its incidence increases over time.^[14]^ It is 4 to 6 times more common in women. The most common type is papillary thyroid cancer, constituting 80% to 85% of all thyroid cancers.^[15]^ In our study, it was 4.2 times more common in women and 90.9% constituted papillary thyroid cancer and is consistent with the literature. In addition, a statistically significant relationship was found between multifocal thyroid cancers and invasion, and previous studies showed a relationship between multifocality and invasion.^[16]^ It was observed that the risk of invasion increased as the tumor size increased and the prognosis of the disease worsened, which supports other previous studies.^[17]^

The systemic inflammatory response can suppress the antitumor activity of immune cells. Neutrophilia affects the immune system by suppressing the cytolytic capability of lymphocytes, activated T cells, and natural killer cells.^[18]^ Monocytes suppress lymphocyte activation and activate tumorigenesis by stimulating tumor neovasculogenesis.^[19]^ And platelet activation plays an important role in malignant cell transformation and metastasis.^[20]^ As a result, it has been observed that surrogate markers for systemic inflammation, which are expressed as increased neutrophil, monocyte, and platelet counts, and decreased lymphocyte count, are associated with the prognosis of various cancers, either alone or as their ratio.^[18–22]^

In the literature, some studies have shown that NLR, PLR, and LMR are associated with thyroid cancers^[23–25]^ and NLR and PLR are also associated with tumor size and lymph node metastasis.^[13,24]^ However, in other studies, no relationship was found between NLR, PLR, and thyroid cancers.^[20,26,27]^ The relationship between inflammatory markers and thyroid cancers is still controversial. In the present study, no significant relationship was found between NLR, LMR, PLR and tumor size, invasion, and metastasis.

SIRI, a cancer-related inflammatory marker, was developed by Que et al and in a meta-analysis, it was found to be an indicator of poor prognosis in many cancer types.^[12,28]^ In a study conducted by Xie et al,^[29]^ including 95 patients with papillary thyroid cancer, SIRI was found to be an important risk factor. In our study, having 340 patients with thyroid cancer, we did not find a significant relationship between SIRI and tumor size, metastasis, and invasion.

The SII, another prognostic marker, was found to be higher in patients with thyroid cancer compared to healthy individuals in a study, but no correlation was found between lymph node involvement, invasion, and tumor size.^[30]^ However, in another study, a relationship was found between lymph node involvement and SII.^[31]^ In our study, no significant relationship was found between SII and tumor size, metastasis, and invasion in thyroid cancer patients.

Another inflammatory marker found to be a prognostic indicator in patients developing acute renal failure after major surgery by Gameiro et al^[10]^ is N/LP. In the literature, no study was found showing the relationship between N/LP and thyroid cancer and other cancers. In our study, N/LP increased significantly with increasing tumor size (OR: 1.4, 95% CI: 1.0–2.0, *P*: .003). Consistent with the literature, an increase in neutrophil and thrombocyte counts and a decrease in lymphocyte count were also observed in our study.^[17–21]^ However, the platelet count did not increase to the expected value, because cancer cells cause both platelet activation and thrombosis in in vivo and in vitro studies.^[32]^ Due to the possibility of thrombus formation, platelet count was lower than expected and the N/LP was increased significantly. Also, it was documented that hematology parameters in healthy men have significant systemic 24-hour variations with different peak times. Peak time in neutrophils occurs approximately 4 hours earlier than that of lymphocytes^[33]^ whereas platelets peaks at around 2 to 3 pm^[34,35]^ In cancers, in thyroid cancer, particularly normal circadian rhythms can be disturbed.^[36]^ As a result of tumor progression may change 24-h patterns of hematology parameters differentially.

Although this study is a retrospective study and therefore other causes of thrombocytopenia and neutrophilia (drug, infection, autoimmunity)^[37,38]^ cannot be excluded, it resulted in outliers that were ignored at the time the sample was taken. But N/LP is thought to be a new inflammatory marker in evaluating the prognosis of cancer diseases.

The main limitations are that a single-center study and retrospective nature of the study, which made it difficult to exclude other major factors that could have influenced inflammatory markers, such as infections, other noninfectious diseases, drugs, etc. Cheap, simple, and effective markers are being developed to determine the prognosis of thyroid cancer as with many cancer types. To the best of our knowledge, the strength of our study is the evaluation of unused markers in addition to previously used inflammatory markers in a large patient population.

There is a relationship between multifocality, and invasion and the risk of invasion increases as the tumor size increases in thyroid cancer. The N/LP ratio was significant as it could be a new marker in showing the relationship between thyroid cancer and its prognosis. Further studies are needed in which the prognosis is followed up, longer-term, more comprehensive, and confounding factors are excluded.

## Author contributions

**Conceptualization:** Seval Müzeyyen Ecin, Deniz Gezer.

**Data curation:** Deniz Gezer.

**Formal analysis:** Seval Müzeyyen Ecin.

**Methodology:** Seval Müzeyyen Ecin.

**Resources:** Seval Müzeyyen Ecin.

**Supervision:** Seval Müzeyyen Ecin, Deniz Gezer.

**Validation:** Seval Müzeyyen Ecin.

**Visualization:** Deniz Gezer.

**Writing – original draft:** Seval Müzeyyen Ecin.

**Writing – review & editing:** Seval Müzeyyen Ecin.
